# A New Efficient Expression for the Conditional Expectation of the Blind Adaptive Deconvolution Problem Valid for the Entire Range ofSignal-to-Noise Ratio

**DOI:** 10.3390/e21010072

**Published:** 2019-01-15

**Authors:** Monika Pinchas

**Affiliations:** Department of Electrical and Electronic Engineering, Ariel University, Ariel 40700, Israel; monika.pinchas@gmail.com; Tel.: +972-524280075; Fax: +972-39066238

**Keywords:** Bayesian approach, deconvolution, maximum entropy density approximation technique

## Abstract

In the literature, we can find several blind adaptive deconvolution algorithms based on closed-form approximated expressions for the conditional expectation (the expectation of the source input given the equalized or deconvolutional output), involving the maximum entropy density approximation technique. The main drawback of these algorithms is the heavy computational burden involved in calculating the expression for the conditional expectation. In addition, none of these techniques are applicable for signal-to-noise ratios lower than 7 dB. In this paper, I propose a new closed-form approximated expression for the conditional expectation based on a previously obtained expression where the equalized output probability density function is calculated via the approximated input probability density function which itself is approximated with the maximum entropy density approximation technique. This newly proposed expression has a reduced computational burden compared with the previously obtained expressions for the conditional expectation based on the maximum entropy approximation technique. The simulation results indicate that the newly proposed algorithm with the newly proposed Lagrange multipliers is suitable for signal-to-noise ratio values down to 0 dB and has an improved equalization performance from the residual inter-symbol-interference point of view compared to the previously obtained algorithms based on the conditional expectation obtained via the maximum entropy technique.

## 1. Introduction

In this paper, the blind adaptive deconvolution problem is addressed, which arises in many applications, such as seismology, underwater acoustic, image restoration and digital communication [1,2,3,4,5,6,7,8,9,10,11,12,13,14,15,16,17,18,19,20,21,22,23,24,25,26,27,28,29,30,31,32,33,34,35]. In digital communication applications, the problem is often called blind adaptive equalization. Non-blind adaptive equalizers, unlike blind adaptive equalizers, require training symbols to generate the error that is fed into the adaptive mechanism which updates the equalizer’s taps. Therefore, blind adaptive equalizers have some important advantages compared with the non-blind version: (1) Simplified protocols in point-to-point communications, avoiding the retransmission of training symbols after abrupt changes of the channel. (2) Higher bandwidth efficiency in broadcast networks. (3) Reduced interoperability problems derived from the use of different training symbols. It is well known that inter-symbol interference (ISI) is a limiting factor in many communication environments, where it causes an irreducible degradation of the bit error rate thus imposing an upper limit on the data symbol rate [36]. In order to overcome the ISI problem, an equalizer is implemented in those systems [34,35,36,37,38,39,40,41]. In this work, the T-spaced blind adaptive equalizer is considered for the single-input-single-output (SISO) case where the sampling rate is equal to the symbol rate (thus referred to as T-spaced where T denotes the baud, or symbol, duration). Please note that a fractionally-spaced equalizer (FSE) where the sampling rate is higher than the symbol rate can be modeled with a single-input-multiple-output (SIMO) system. In addition, a SIMO system can be modeled with a parallel combination of T-spaced blind adaptive equalizers. Thus, improving the equalization performance of a T-spaced blind adaptive equalizer for the SISO case may also lead to equalization performance improvement for the SIMO case. As already mentioned above, blind adaptive equalizers do not use any training symbols to generate the error that is fed into the adaptive mechanism which updates the equalizer’s taps. Instead of those training symbols, an estimate of the desired response is obtained via the use of a nonlinear transformation to sequences involved in the adaptation process. Now, very often, blind adaptive equalization algorithms are classified according to the location of their nonlinearity in the algorithm chain [42]. According to Reference [42], there are three different types: (1) polyspectral algorithms, (2) Bussgang-type algorithms, (3) probabilistic algorithms. In the first method, the nonlinearity is located at the output of the channel, right before the equalizer. Thus, the nonlinearity actually estimates the channel. This estimation is fed into the adaptive mechanism which updates the equalizer’s taps. In the second type, the nonlinearity is situated at the output of the equalizer. Here, the nonlinearity can just be the estimation of the source signal via the the use of the conditional expectation (the expectation of the source input given the equalized or deconvolutional output), or the nonlinearity can be a predefined cost function that holds some information of the ISI. Thus, minimizing this predefined cost function with respect to the equalizer’s taps may lower the residual ISI and help in the symbol recovery process. Since Bussgang-type algorithms often have shorter convergence times than polyspectral methods, which need larger amounts of data for an equivalent estimation variance, they are more popular [42]. In the third class of algorithms, directly locating the nonlinearity is more problematic compared to the first two groups since the nonlinearity is combined with the data detection process. While these algorithms can extract considerable information from relatively little data, this is often accomplished at a huge computational cost [42]. In the following, the Bussgang-type blind equalization algorithms are considered, where the conditional expectation (the expectation of the source input given the equalized or deconvolutional output) is derived for estimating the desired response. In the literature, we can find several approximated expressions for the conditional expectation related to the blind adaptive deconvolutional problem [20,43,44,45,46,47,48,49]. However, References [43,44,45,46] are valid only for a uniformly distributed source input and References [20,47,48] were designed only for the noiseless case. Recently [49], a new blind adaptive equalization method was proposed based on Reference [47] that is applicable for signal-to-noise ratio (SNR) values down to 7 dB. However, the computational burden of the method in Reference [49] is relative high. The closed-form approximated expression proposed in Reference [49] for the conditional expectation with Lagrange multipliers up to order four (thus applicable for the 16QAM input case) is composed of a polynomial function of the equalized output of order twenty one. Please note that the proposed expression for the conditional expectation with Lagrange multipliers up to order four in Reference [47] is a polynomial function of the equalized output of order thirteen while the proposed expression for the conditional expectation with Lagrange multipliers up to order four in Reference [20] is a fraction where the numerator and the denominator are a polynomial function of the equalized output of order thirteen and twelve, respectively.

In this paper, I propose a new closed-form approximated expression for the conditional expectation based on Reference [20] with Lagrange multipliers up to order four. This new proposed expression has a reduced computational burden compared with the previously obtained expressions for the conditional expectation proposed in Reference [20,47,49]. The new proposed expression for the conditional expectation is composed of a fraction where the numerator and the denominator are a polynomial function of the equalized output of order seven and six, respectively. Simulation results show that the new proposed algorithm, with the new proposed Lagrange multipliers up to order four, is suitable for SNR values down to 0 dB and has an improved equalization performance from the residual ISI point of view compared with Reference [49].

## 2. System Description

**Figure 1:**
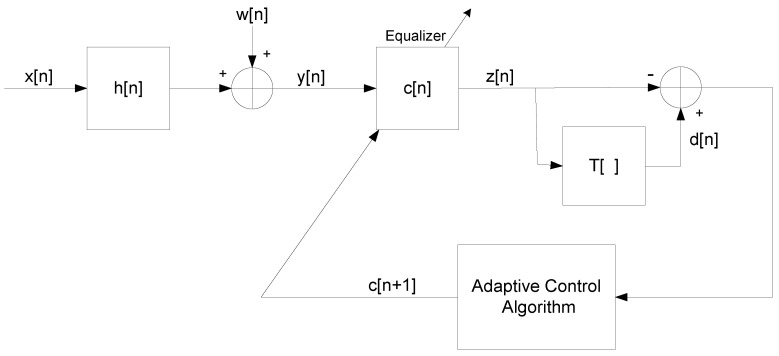
Block diagram of the system.

I consider the system from References [20,47,49], illustrated in Figure 1, where I make the following assumptions as were done in References [20,47,49]:

1. The source signal x[n] is given by
  (1) $$x\left[n\right]=x_{1}\left[n\right]+jx_{2}\left[n\right]$$
  where $x_{1}\left[n\right]$ and $x_{2}\left[n\right]$ are the real and imaginary parts of x[n], respectively. It is assumed that $x_{1}\left[n\right]$ and $x_{2}\left[n\right]$ are independent and that
  (2) E[x[n]]=0
  where E[·] stands for the expectation operation.
2. The unknown channel h[n] is possibly a non-minimum phase linear time-invariant filter in which the transfer function has no “deep zeros”.
3. The filter c[n] is a tap-delay line.
4. The channel noise w[n] is an additive Gaussian white noise with variance $\sigma_{w}^{2}$ where $\sigma_{w_{r}}^{2}$ and $\sigma_{w_{i}}^{2}$ are the variances of the real and imaginary parts of w[n], respectively.
5. The function T[·] is a memoryless nonlinear function that satisfies the additivity condition:
  (3) $$T\left[z_{1}\left[n\right]+jz_{2}\left[n\right]\right]=T\left[z_{1}\left[n\right]\right]+jT\left[z_{2}\left[n\right]\right]$$
  where $z_{1}\left[n\right]$, $z_{2}\left[n\right]$ are the real and imaginary parts of the equalized output, respectively.

As was described in References [20,47,49], the source input x[n] is sent via the channel h[n] and is corrupted with channel noise w[n]. The ideal equalized output is expressed in Reference [50] as

(4) $$z\left[n\right]=x\left[n-D\right]e^{j\theta }$$

where *D* is a constant delay and θ is a constant phase shift. Therefore, in the ideal case, we could write

(5) $$c\left[n\right]*h\left[n\right]=\delta \left[n-D\right]e^{j\theta }$$

where “*” denotes the convolution operation and δ is the Kronecker delta function. In this paper, I assume, as was also done in References [20,47,49], that *D* and θ are equal to zero (please refer to Reference [49] for the explanation). The equalized output is given by

(6) $$z\left[n\right]=x\left[n\right]+\tilde{p}\left[n\right]+\tilde{w}\left[n\right]$$

where $\tilde{p}\left[n\right]$ is the convolutional noise and

(7) $$\tilde{w}\left[n\right]=w\left[n\right]*c\left[n\right].$$

In this paper, the ISI is used to measure the performance of the deconvolution process, defined by

(8) $$ISI=\frac{\sum_{\tilde{m}}|\tilde{s}\left[\tilde{m}\right]{|}^{2}-{\left|\tilde{s}\right|}_{max}^{2}}{|\tilde{s}{|}_{max}^{2}}$$

where $|\tilde{s}{|}_{max}$ is the component of $\tilde{s}$, given in (8), having the maximal absolute value.

(9) $$\tilde{s}\left[n\right]=c\left[n\right]*h\left[n\right]=\delta \left[n\right]+\xi \left[n\right]$$

where ξ[n] stands for some error not having the ideal case. The input sequence x[n] is estimated with the function T[z[n]] and is denoted as d[n]. The difference between T[z[n]] and the equalized output $z\left[n\right]$ is denoted as $\tilde{e}\left[n\right]$:

(10) $$\tilde{e}\left[n\right]=T\left[z\left[n\right]\right]-z\left[n\right]$$

This error plays an important role in updating the equalizer’s taps:

(11) $$\underline{c}\left[n+1\right]=\underline{c}\left[n\right]+\mu \tilde{e}\left[n\right]{\underline{y}}^{*}\left[n\right]$$

where ${\left(\cdot \right)}^{*}$ is the conjugate operation on (·), μ is the step size parameter, and $\underline{c}\left[n\right]$ is the equalizer vector, where the input vector is $\underline{y}\left[n\right]={\left[y\left[n\right]\ldots y\left[n-N+1\right]\right]}^{T}$. The operator ${\left(\right)}^{T}$ denotes the transpose of the function (), and *N* is the equalizer’s tap length. Another way to update the equalizer’s taps is to use the cost function approach [51]:

(12) $$\underline{c}\left[n+1\right]=\underline{c}\left[n\right]-\mu \frac{\partial F[n]}{\partial z[n]}{\underline{y}}^{*}\left[n\right]$$

where F[n] is a predefined function that characterizes the ISI. In the literature [20,47,49,50], we may find the conditional expectation (E[x[n]|z[n]]) as a proper option for T[z[n]]. According to Reference [20], the conditional expectation for the real valued and noiseless case was obtained via Bayes rules:

(13) $$E\left[x[n]|z[n]\right]=\frac{\int_{-\infty }^{\infty }x\left[n\right]f_{z|x}\left(z|x\right)f_{x}\left(x\right)dx}{f_{z}\left(z\right)}$$

where $f_{z|x}\left(z|x\right)$ was given by

(14) $$\begin{array}{c} f_{z|x}\left(z|x\right)=\frac{1}{\sqrt{2\pi }\sigma_{\tilde{p}}}\exp\left(-\frac{{\left(z[n]-x[n]\right)}^{2}}{2\sigma_{\tilde{p}}^{2}}\right) \end{array}$$

and the source probability density function (pdf) ($f_{x}\left(x\right)$) was approximated by the maximum entropy density approximation technique:

(15) $$\begin{array}{c} {\hat{f}}_{x}\left(x\right)=\exp\left(\sum_{k=0}^{K}{\hat{\lambda }}_{k}x^{k}\left[n\right]\right) \end{array}$$

where ${\hat{f}}_{x}\left(x\right)$ is the approximated probability density function and ${\hat{\lambda }}_{k}$ (k=0,1,2,…,K) are the Lagrange multipliers. In the following, for simplicity, I write *x* and *z* instead of x[n] and z[n], respectively. By using (13)–(15), the conditional expectation obtained by Reference [20] for the real valued and noiseless case is

(16) $$\begin{array}{c} E\left[x|z\right]=\frac{z+\frac{{\hat{g}}_{1}^{\prime \prime }\left(z\right)}{2\hat{g}\left(z\right)}\sigma_{\tilde{p}}^{2}+\frac{{\hat{g}}_{1}^{\left(4\right)}\left(z\right)}{8\hat{g}\left(z\right)}{\left(\sigma_{\tilde{p}}^{2}\right)}^{2}}{1+\frac{{\hat{g}}^{\prime \prime }\left(z\right)}{2\hat{g}\left(z\right)}\sigma_{\tilde{p}}^{2}+\frac{{\hat{g}}^{\left(4\right)}\left(z\right)}{8\hat{g}\left(z\right)}{\left(\sigma_{\tilde{p}}^{2}\right)}^{2}} \end{array}$$

where

(17) $$\begin{array}{c} \hat{g}\left(z\right)={\left\{\exp\left(\sum_{k=2}^{k=K}{\hat{\lambda }}_{k}x^{k}\right)\right\}}_{x=z}\\ \;{\hat{g}}^{\prime \prime }\left(z\right)={\left\{\frac{d^{2}}{dx^{2}}\left[\exp\left(\sum_{k=2}^{k=K}{\hat{\lambda }}_{k}x^{k}\right)\right]\right\}}_{x=z};\;{\hat{g}}^{\left(4\right)}\left(z\right)={\left\{\frac{d^{4}}{dx^{4}}\left[\exp\left(\sum_{k=2}^{k=K}{\hat{\lambda }}_{k}x^{k}\right)\right]\right\}}_{x=z}\\ \;{\hat{g}}_{1}^{\prime \prime }\left(z\right)={\left\{\frac{d^{2}}{dx^{2}}\left[x\exp\left(\sum_{k=2}^{k=K}{\hat{\lambda }}_{k}x_{s}^{k}\right)\right]\right\}}_{x=z};\;{\hat{g}}_{1}^{\left(4\right)}\left(z\right)={\left\{\frac{d^{4}}{dx^{4}}\left[x\exp\left(\sum_{k=2}^{k=K}{\hat{\lambda }}_{k}x^{k}\right)\right]\right\}}_{x=z} \end{array}$$

and

(18) $$\sigma_{\tilde{p}}^{2}=E\left[{\tilde{p}}^{2}\left[n\right]\right].$$

Please note that for ${\hat{\lambda }}_{k}$ up to order four, ${\hat{g}}^{\prime \prime }\left(z\right)$, ${\hat{g}}_{1}^{\prime \prime }\left(z\right)$, ${\hat{g}}^{\left(4\right)}\left(z\right)$, and ${\hat{g}}_{1}^{\left(4\right)}\left(z\right)$ are polynomial functions of order seven, six, thirteen, and twelve, respectively. According to Reference [20], the Lagrange multipliers were obtained by minimizing the approximated obtained mean square error (MSE) [20] with respect to the Lagrange multipliers. Namely, the Lagrange multipliers were obtained by

(19) $$\begin{array}{c} \frac{d}{d{\hat{\lambda }}_{k}}\left(E\left[{\left(\hat{x}-x\right)}^{2}\right]\right)=0\\ \;\;\;⇓\\ m_{k-2}\left(k-1\right)k+2{\hat{\lambda }}_{k}m_{2k-2}k^{2}+\sum_{L=3,L\neq k}^{K}2{\hat{\lambda }}_{L}m_{k+L-2}kL=0\;\;\mathrm{for}\;k=2,4,6,\ldots ,K \end{array}$$

where

(20) $$m_{G}=E\left[x^{G}\right],$$

$\hat{x}$ is the conditional expectation, $E\left[{\left(\hat{x}-x\right)}^{2}\right]$ is the MSE and given by Reference [20] as

(21) $$\begin{array}{c} E\left[{\left(\hat{x}-x\right)}^{2}\right]≃\sigma_{\tilde{p}}^{2}\left(1-\sigma_{\tilde{p}}^{2}\left(2E\left[\frac{{\hat{g}}^{{}^{\prime \prime }}\left(x\right)}{2\hat{g}\left(x\right)}\right]\right)\right). \end{array}$$

For the Lagrange multipliers ${\hat{\lambda }}_{k}$ up to order four, based on (19), we obtain the following equations for ${\hat{\lambda }}_{2}$ and ${\hat{\lambda }}_{4}$:

(22) $$\begin{array}{c} 2+2{\hat{\lambda }}_{2}m_{2}4+2{\hat{\lambda }}_{4}m_{4}8=0\\ m_{2}12+2{\hat{\lambda }}_{4}m_{6}16+2{\hat{\lambda }}_{2}m_{4}8=0 \end{array}$$

## 3. The New Proposed Expression for the Conditional Expectation

In this section, I present my newly proposed approximated closed-form expression for the conditional expectation based on Reference [20]. In the following, I adopt the assumptions made in References [20,47,49]:

1. The convolutional noise $\tilde{p}\left[n\right]$ is a zero mean, white Gaussian process with variance
  (23) $$\sigma_{\tilde{p}}^{2}=E\left[\tilde{p}\left[n\right]{\tilde{p}}^{*}\left[n\right]\right].$$
2. The source signal x[n] is an independent non-Gaussian signal with known variance and higher moments.
3. The convolutional noise $\tilde{p}\left[n\right]$ and the source signal are independent.
4. The convolutional noise power $\sigma_{\tilde{p}}^{2}$ is sufficiently low.

For justification of the above assumptions, please refer to Reference [49]. In the following, I first consider the real valued case and then turn back to the case where the real and imaginary parts of the input signal are independent (as is the case for the 16QAM source input). According to (19) and (21), we may see that the obtained Lagrange multipliers are depending only on the second leading term associated to the denominator of (16). This may imply that we can use a truncated version of (16) for the approximated conditional expectation expression where the computational burden is automatically reduced compared to (16).

**Proposition** **1.** *In this paper, I propose for the real valued and noisy case the following expression for the conditional expectation with Lagrange multipliers up to order four:*

(24) $$\begin{array}{c} E\left[x|z\right]=\frac{z+\frac{{\hat{g}}_{1}^{\prime \prime }\left(z\right)}{2\hat{g}\left(z\right)}\sigma_{p}^{2}}{1+\frac{{\hat{g}}^{\prime \prime }\left(z\right)}{2\hat{g}\left(z\right)}\sigma_{p}^{2}} \end{array}$$

*where*

(25) $$\begin{array}{c} \sigma_{p}^{2}=\sigma_{\tilde{p}}^{2}+\sigma_{\tilde{w}}^{2}\\ \frac{{\hat{g}}_{1}^{{}^{\prime \prime }}\left(z\right)}{2\hat{g}\left(z\right)}=z\left(8z^{6}\lambda_{4}^{2}+8z^{4}\lambda_{2}\lambda_{4}+2z^{2}\lambda_{2}^{2}+10z^{2}\lambda_{4}+3\lambda_{2}\right)\\ \frac{{\hat{g}}^{{}^{\prime \prime }}\left(z\right)}{2\hat{g}\left(z\right)}=8z^{6}\lambda_{4}^{2}+8z^{4}\lambda_{2}\lambda_{4}+2z^{2}\lambda_{2}^{2}+6z^{2}\lambda_{4}+\lambda_{2} \end{array}$$

*and*

(26) $$\begin{array}{c} \lambda_{2}=\frac{1}{4{\tilde{m}}_{2}\left(64{\tilde{m}}_{4}^{2}-64{\tilde{m}}_{2}{\tilde{m}}_{6}\right)}\left(64{\tilde{m}}_{2}{\tilde{m}}_{6}-64{\tilde{m}}_{4}^{2}+8{\tilde{m}}_{4}\left(8{\tilde{m}}_{4}-24{\tilde{m}}_{2}^{2}\right)\right)\\ \lambda_{4}=-\frac{1}{64{\tilde{m}}_{4}^{2}-64{\tilde{m}}_{2}{\tilde{m}}_{6}}\left(8{\tilde{m}}_{4}-24{\tilde{m}}_{2}^{2}\right)\\ \;\;with\\ {\tilde{m}}_{2}=m_{2}\left(1+\frac{1}{SNR\sum_{k=0}^{R-1}{\left|h_{k}\right|}^{2}}\right)\\ {\tilde{m}}_{4}=m_{2}^{2}\left(\frac{3}{{\left(SNR\sum_{k=0}^{R-1}{\left|h_{k}\right|}^{2}\right)}^{2}}+\frac{6}{SNR\sum_{k=0}^{R-1}{\left|h_{k}\right|}^{2}}+\frac{m_{4}}{m_{2}^{2}}\right)\\ {\tilde{m}}_{6}=m_{2}^{3}\left(\frac{15}{{\left(SNR\sum_{k=0}^{R-1}{\left|h_{k}\right|}^{2}\right)}^{3}}+\frac{45}{{\left(SNR\sum_{k=0}^{R-1}{\left|h_{k}\right|}^{2}\right)}^{2}}+\frac{15}{SNR\sum_{k=0}^{R-1}{\left|h_{k}\right|}^{2}}\frac{m_{4}}{m_{2}^{2}}+\frac{m_{6}}{m_{2}^{3}}\right) \end{array}$$

where *R* is the channel tap length, $h_{k}$ is the k-th tap of the channel h[n], and

(27) $$SNR=\frac{m_{2}}{\sigma_{w}^{2}};\;\;\;\sigma_{\tilde{w}}^{2}=E\left[{\tilde{w}}^{2}\right].$$

**Proof** **of the proposed Lagrange multipliers given in (26).** According to Reference [20] (Appendix B), the approximated MSE for the noisy case, valid at the latter stages of the deconvolutional process, may be given as

(28) $$\begin{array}{c} E\left[{\left(\hat{x}-x\right)}^{2}\right]≃\sigma_{p}^{2}\left(1-\sigma_{p}^{2}\left(2E\left[\frac{{\hat{g}}^{{}^{\prime \prime }}\left(\tilde{x}\right)}{2\hat{g}\left(\tilde{x}\right)}\right]\right)\right) \end{array}$$

where

(29) $$\tilde{x}=x+\tilde{w}.$$

Thus, according to (19), we obtain the following equations for the Lagrange multipliers:

(30) $$\begin{array}{c} 2+2\lambda_{2}{\tilde{m}}_{2}4+2\lambda_{4}{\tilde{m}}_{4}8=0\\ {\tilde{m}}_{2}12+2\lambda_{4}{\tilde{m}}_{6}16+2\lambda_{2}{\tilde{m}}_{4}8=0 \end{array}$$

where

(31) $${\tilde{m}}_{G}=E\left[{\tilde{x}}^{G}\right].$$

The solution of (30) for $\lambda_{2}$ and $\lambda_{4}$ as a function of ${\tilde{m}}_{2}$, ${\tilde{m}}_{4}$, and ${\tilde{m}}_{6}$ is given in (26). Next, I find closed-form approximated expressions for ${\tilde{m}}_{2}$, ${\tilde{m}}_{4}$, and ${\tilde{m}}_{6}$. When the deconvolutional process has converged and leaves the system with a convolutional noise that can be considered as very low, we may write, according to Reference [52],

(32) $$\sigma_{\tilde{w}}^{2}=\frac{\sigma_{w}^{2}}{\sum_{k=0}^{R-1}{\left|h_{k}\right|}^{2}}.$$

Since *x* and *w* are independent, by using (32) and the expression for SNR (27) we have

(33) $$\begin{array}{c} {\tilde{m}}_{2}=E\left[{\left(x+\tilde{w}\right)}^{2}\right]=m_{2}+\sigma_{\tilde{w}}^{2}=m_{2}+\frac{\sigma_{w}^{2}}{\sum_{k=0}^{R-1}{\left|h_{k}\right|}^{2}}=m_{2}\left(1+\frac{\sigma_{w}^{2}}{m_{2}\sum_{k=0}^{R-1}{\left|h_{k}\right|}^{2}}\right)=\\ m_{2}\left(1+\frac{1}{SNR\sum_{k=0}^{R-1}{\left|h_{k}\right|}^{2}}\right)\\ {\tilde{m}}_{4}=E\left[{\left(x+\tilde{w}\right)}^{4}\right]=3\sigma_{\tilde{w}}^{4}+6m_{2}\sigma_{\tilde{w}}^{2}+m_{4}=3{\left(\frac{\sigma_{w}^{2}}{\sum_{k=0}^{R-1}{\left|h_{k}\right|}^{2}}\right)}^{2}+6m_{2}\frac{\sigma_{w}^{2}}{\sum_{k=0}^{R-1}{\left|h_{k}\right|}^{2}}+m_{4}=\\ m_{2}^{2}\left(\frac{3}{{\left(SNR\sum_{k=0}^{R-1}{\left|h_{k}\right|}^{2}\right)}^{2}}+\frac{6}{SNR\sum_{k=0}^{R-1}{\left|h_{k}\right|}^{2}}+\frac{m_{4}}{m_{2}^{2}}\right)\\ {\tilde{m}}_{6}=E\left[{\left(x+\tilde{w}\right)}^{6}\right]=15\sigma_{\tilde{w}}^{6}+45\sigma_{\tilde{w}}^{4}m_{2}+15\sigma_{\tilde{w}}^{2}m_{4}+m_{6}=\\ m_{2}^{3}\left(\frac{15}{{\left(SNR\sum_{k=0}^{R-1}{\left|h_{k}\right|}^{2}\right)}^{3}}+\frac{45}{{\left(SNR\sum_{k=0}^{R-1}{\left|h_{k}\right|}^{2}\right)}^{2}}+\frac{15}{SNR\sum_{k=0}^{R-1}{\left|h_{k}\right|}^{2}}\frac{m_{4}}{m_{2}^{2}}+\frac{m_{6}}{m_{2}^{3}}\right) \end{array}$$

This completes our proof. ▯

Next, I turn to the 16QAM source input. For this case, according to Reference [44], I have that

(34) $$E\left[x|z\right]=E\left[x_{1}|z_{1}\right]+jE\left[x_{2}|z_{2}\right].$$

## 4. Simulation

In this section, I show via simulation results the efficiency of the truncated expression for the conditional expectation (24) combined with the Lagrange multipliers given in (26). Namely, I show via simulation results the equalization performance from the residual ISI point of view of the new proposed algorithm (with (24) and (26)) compared to the simulation results obtained by the maximum entropy [49] method and Godard’s [53] algorithm. Godard’s [53] algorithm is used for comparison since it is a very efficient algorithm from the equalization performance point of view [28]. In addition, it’s computational burden is very low [28]. For Godard’s algorithm [53], the equalizer’s taps were updated as follows:

(35) $$\;c_{l}\left[n+1\right]=c_{l}\left[n\right]-\mu_{G}\left({\left|z\right|}^{2}-\frac{E\left[{\left|x\right|}^{4}\right]}{E\left[{\left|x\right|}^{2}\right]}\right)y^{*}\left[n-l\right]$$

where $\mu_{G}$ is a positive step size parameter and *l* stands for the l-th tap of the equalizer. The update mechanism of the equalizer’s taps associated with the recently obtained maximum entropy algorithm [49] was as follows:

(36) $$\;{\tilde{c}}_{l}\left[n+1\right]=c_{l}\left[n\right]-\mu_{ANEW}Wy^{*}\left[n-l\right]$$

with

(37) $$\;W=\left[\left(E\left[x_{1}|z_{1}\right]\left[\frac{\left(z_{1}\left[n\right]E\left[x_{1}|z_{1}\right]\right)}{{\langle {\left(z_{1}\right)}^{2}\rangle }_{n}}\right]+jE\left[x_{2}|z_{2}\right]\left[\frac{\left(z_{2}\left[n\right]E\left[x_{2}|z_{2}\right]\right)}{{\langle {\left(z_{2}\right)}^{2}\rangle }_{n}}\right]\right)-z\left[n\right]\right]$$

and

(38) $$\begin{array}{c} E\left[x_{1}|z_{1}\right]≃\left(1+\left(\varepsilon_{0}^{1}+\varepsilon_{2}^{1}z_{1}^{2}+\varepsilon_{4}^{1}z_{1}^{4}\right)+\frac{1}{2}{\left(\varepsilon_{0}^{1}+\varepsilon_{2}^{1}z_{1}^{2}+\varepsilon_{4}^{1}z_{1}^{4}\right)}^{2}\right)\left(z_{1}+\frac{\sigma_{p_{1}}^{2}}{2}\frac{g_{1}^{{}^{\prime \prime }}\left(z_{1}\right)}{g(z_{1})}+\frac{{\left(\sigma_{p_{1}}^{2}\right)}^{2}}{8}\frac{g_{1}^{{}^{{}^{⁗}}}\left(z_{1}\right)}{g(z_{1})}\right)\\ E\left[x_{2}|z_{2}\right]≃\left(1+\left(\varepsilon_{0}^{2}+\varepsilon_{2}^{2}z_{2}^{2}+\varepsilon_{4}^{2}z_{2}^{4}\right)+\frac{1}{2}{\left(\varepsilon_{0}^{2}+\varepsilon_{2}^{2}z_{2}^{2}+\varepsilon_{4}^{2}z_{2}^{4}\right)}^{2}\right)\left(z_{2}+\frac{\sigma_{p_{2}}^{2}}{2}\frac{g_{1}^{{}^{\prime \prime }}\left(z_{2}\right)}{g(z_{2})}+\frac{{\left(\sigma_{p_{2}}^{2}\right)}^{2}}{8}\frac{g_{1}^{{}^{{}^{⁗}}}\left(z_{2}\right)}{g(z_{2})}\right)\\ where\\ s=1,2\\ \frac{g_{1}^{{}^{\prime \prime }}\left(z_{s}\right)}{g(z_{s})}=2z_{s}\left(8z_{s}^{6}\lambda_{4}^{2}+8z_{s}^{4}\lambda_{2}\lambda_{4}+2z_{s}^{2}\lambda_{2}^{2}+10z_{s}^{2}\lambda_{4}+3\lambda_{2}\right)\\ \frac{g_{1}^{{}^{{}^{⁗}}}\left(z_{s}\right)}{g(z_{s})}=4z_{s}\left(64z_{s}^{12}\lambda_{4}^{4}+128z_{s}^{10}\lambda_{2}\lambda_{4}^{3}+96z_{s}^{8}\lambda_{2}^{2}\lambda_{4}^{2}+352z_{s}^{8}\lambda_{4}^{3}+32z_{s}^{6}\lambda_{2}^{3}\lambda_{4}+432z_{s}^{6}\lambda_{2}\lambda_{4}^{2}+\right.\\ \left.4z_{s}^{4}\lambda_{2}^{4}+168z_{s}^{4}\lambda_{2}^{2}\lambda_{4}+348z_{s}^{4}\lambda_{4}^{2}+20z_{s}^{2}\lambda_{2}^{3}+180z_{s}^{2}\lambda_{2}\lambda_{4}+15\lambda_{2}^{2}+30\lambda_{4}\right)\\ \sigma_{p_{s}}^{2}=\sigma_{z_{s}}^{2}-\sigma_{x_{s}}^{2} \end{array}$$

and

(39) $$\sigma_{x_{s}}^{2}=E\left[x_{s}^{2}\right].$$

According to Reference [49],

(40) $$\sigma_{z_{s}}^{2}=E\left[z_{s}^{2}\right],$$

and given by

(41) $$\;\langle z_{s}^{2}\rangle =\left(1-\beta_{ANEW}\right){\langle z_{s}^{2}\rangle }_{n-1}+\beta_{ANEW}{\left(z_{s}\right)}_{n}^{2}$$

where 〈〉 stands for the estimated expectation, ${\langle z_{s}^{2}\rangle }_{0}>0$, $\beta_{ANEW}$ and $\mu_{ANEW}$ are positive step size parameters. $\varepsilon_{0}^{s}$, $\varepsilon_{2}^{s}$, $\varepsilon_{4}^{s}$, $\lambda_{2}$, and $\lambda_{4}$ were set according to Reference [49] as

(42) $$\begin{array}{c} \varepsilon_{0}^{s}=-2\lambda_{2}\sigma_{p_{s}}^{2};\;\;\varepsilon_{2}^{s}=-\sigma_{p_{s}}^{2}\left(4\lambda_{2}^{2}+12\lambda_{4}\right);\;\;\varepsilon_{4}^{s}=-16\lambda_{2}\lambda_{4}\sigma_{p_{s}}^{2} \end{array}$$

(43) $$\begin{array}{c} \lambda_{2}≃\frac{1}{40{\bar{m}}_{2}\left(20\;736{\bar{m}}_{4}^{2}+1280{\bar{m}}_{2}{\bar{m}}_{6}\right)}\left(41\;472{\bar{m}}_{4}^{2}+2560{\bar{m}}_{2}{\bar{m}}_{6}-144{\bar{m}}_{4}\left(480{\bar{m}}_{2}^{2}+288{\bar{m}}_{4}\right)\right)\\ \lambda_{4}≃\frac{1}{20\;736{\bar{m}}_{4}^{2}+1280{\bar{m}}_{2}{\bar{m}}_{6}}\left(480{\bar{m}}_{2}^{2}+288{\bar{m}}_{4}\right) \end{array}$$

where

(44) $$E\left[x_{1}^{G}\right]={\bar{m}}_{G}.$$

In order to get an equalization gain of one, the following gain control was used according to Reference [49]:

(45) $$c_{l}\left[n\right]=\frac{{\tilde{c}}_{l}}{\sqrt{\sum_{l}{\left|{\tilde{c}}_{l}\right|}^{2}}}$$

where $c_{l}\left[n\right]$ is the vector of taps after iteration and $c_{l}\left[0\right]$ is some reasonable initial guess. The update mechanism of the equalizer’s taps associated with the new proposed blind equalization method involving the truncated version for the conditional expectation and the newly derived Lagrange multipliers was as follows:

(46) $$\;{\tilde{c}}_{l}\left[n+1\right]=c_{l}\left[n\right]-\mu_{BNEW}Wy^{*}\left[n-l\right]$$

with *W* given in (37), but with

(47) $$\begin{array}{c} E\left[x_{1}|z_{1}\right]=\frac{z_{1}+\frac{{\hat{g}}_{1}^{\prime \prime }\left(z_{1}\right)}{2\hat{g}\left(z_{1}\right)}\sigma_{p_{1}}^{2}}{1+\frac{{\hat{g}}^{\prime \prime }\left(z_{1}\right)}{2\hat{g}\left(z_{1}\right)}\sigma_{p_{1}}^{2}}\\ E\left[x_{2}|z_{2}\right]=\frac{z_{2}+\frac{{\hat{g}}_{1}^{\prime \prime }\left(z_{2}\right)}{2\hat{g}\left(z_{2}\right)}\sigma_{p_{2}}^{2}}{1+\frac{{\hat{g}}^{\prime \prime }\left(z_{2}\right)}{2\hat{g}\left(z_{2}\right)}\sigma_{p_{2}}^{2}}\\ where\\ s=1,2\\ \frac{{\hat{g}}_{1}^{{}^{\prime \prime }}\left(z_{s}\right)}{2\hat{g}\left(z_{s}\right)}=z_{s}\left(8z_{s}^{6}\lambda_{4}^{2}+8z_{s}^{4}\lambda_{2}\lambda_{4}+2z_{s}^{2}\lambda_{2}^{2}+10z_{s}^{2}\lambda_{4}+3\lambda_{2}\right)\\ \frac{{\hat{g}}^{{}^{\prime \prime }}\left(z_{s}\right)}{2\hat{g}\left(z_{s}\right)}=8z_{s}^{6}\lambda_{4}^{2}+8z_{s}^{4}\lambda_{2}\lambda_{4}+2z_{s}^{2}\lambda_{2}^{2}+6z_{s}^{2}\lambda_{4}+\lambda_{2}\\ \sigma_{p_{s}}^{2}=\sigma_{z_{s}}^{2}-\sigma_{x_{s}}^{2} \end{array}$$

(48) $$\begin{array}{c} \lambda_{2}=\frac{1}{4{\hat{m}}_{2}\left(64{\hat{m}}_{4}^{2}-64{\hat{m}}_{2}{\hat{m}}_{6}\right)}\left(64{\hat{m}}_{2}{\hat{m}}_{6}-64{\hat{m}}_{4}^{2}+8{\hat{m}}_{4}\left(8{\hat{m}}_{4}-24{\hat{m}}_{2}^{2}\right)\right)\\ \lambda_{4}=-\frac{1}{64{\hat{m}}_{4}^{2}-64{\hat{m}}_{2}{\hat{m}}_{6}}\left(8{\hat{m}}_{4}-24{\hat{m}}_{2}^{2}\right)\\ \;\;\mathrm{with}\\ {\hat{m}}_{2}={\bar{m}}_{2}\left(1+\frac{1}{SNR\sum_{k=0}^{R-1}{\left|h_{k}\right|}^{2}}\right)\\ {\hat{m}}_{4}={\bar{m}}_{2}^{2}\left(\frac{3}{{\left(SNR\sum_{k=0}^{R-1}{\left|h_{k}\right|}^{2}\right)}^{2}}+\frac{6}{SNR\sum_{k=0}^{R-1}{\left|h_{k}\right|}^{2}}+\frac{{\bar{m}}_{4}}{{\bar{m}}_{2}^{2}}\right)\\ {\hat{m}}_{6}={\bar{m}}_{2}^{3}\left(\frac{15}{{\left(SNR\sum_{k=0}^{R-1}{\left|h_{k}\right|}^{2}\right)}^{3}}+\frac{45}{{\left(SNR\sum_{k=0}^{R-1}{\left|h_{k}\right|}^{2}\right)}^{2}}+\frac{15}{SNR\sum_{k=0}^{R-1}{\left|h_{k}\right|}^{2}}\frac{{\bar{m}}_{4}}{{\bar{m}}_{2}^{2}}+\frac{{\bar{m}}_{6}}{{\bar{m}}_{2}^{3}}\right) \end{array}$$

where

(49) $$SNR=\frac{E[xx^{*}]}{\sigma_{w}^{2}}=\frac{{\bar{m}}_{2}}{\sigma_{w_{r}}^{2}}$$

$\sigma_{z_{s}}^{2}$ was estimated by

(50) $$\;\langle z_{s}^{2}\rangle =\left(1-\beta_{BNEW}\right){\langle z_{s}^{2}\rangle }_{n-1}+\beta_{BNEW}{\left(z_{s}\right)}_{n}^{2}$$

where ${\langle z_{s}^{2}\rangle }_{0}>0$, $\beta_{BNEW}$ and $\mu_{BNEW}$ are positive step size parameters. The equalizer’s taps in (46) were updated only if ${\hat{N}}_{s}>\varepsilon$, where ε is a small positive parameter and

(51) $${\hat{N}}_{s}=1+\frac{{\hat{g}}^{\prime \prime }\left(z_{s}\right)}{2\hat{g}\left(z_{s}\right)}\sigma_{p_{s}}^{2}.$$

I also used here a gain control according to (45).

In the following, I denote “$MaxEnt_{ANEW}$”, “Godard”, and “$MaxEnt_{BNEW}$” as the algorithms given in References [49,53], and (46), respectively. For the “$MaxEnt_{ANEW}$” and “$MaxEnt_{BNEW}$” algorithms, I used

(52) $$E\left[z_{s}^{2}\right]=E\left[x_{s}^{2}\right]$$

for initialization.

The following channels were considered:

**Channel 1**: (initial ISI=0.44): Taken according to References [1,49]: $h_{n}=\left\{0\;\mathrm{for}\;n<0;\;-0.4\;\mathrm{for}\;n=0;\;0.84\cdot 0.4^{n-1}\;\mathrm{for}\;n>0\right\}$.

**Channel 2**: Normalized Channel 1 with $\sum_{k}{\left|h_{k}\right|}^{2}=1.317$.

**Channel 3**: Normalized Channel 1 with $\sum_{k}{\left|h_{k}\right|}^{2}=0.768$.

**Figure 2:**
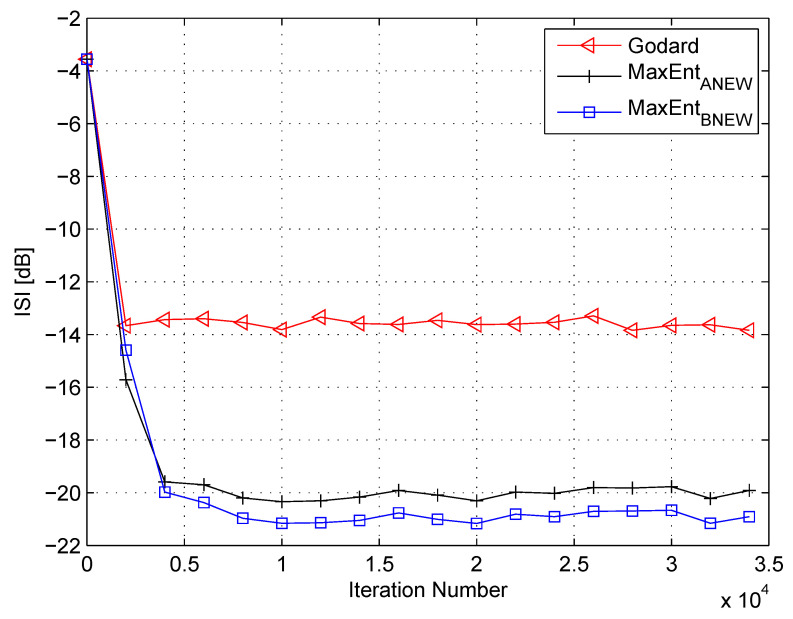
Performance comparison between equalization algorithms for a 16QAM source input going through Channel 1. The averaged results were obtained in 50 Monte Carlo trials for a signal-to-noise ratio (SNR) = 10 dB. $\mu_{G}=7\times 10^{-5}$, $\mu_{ANEW}=0.00009$, $\beta_{ANEW}=1\times 10^{-5}$, $\mu_{BNEW}=0.00008$, $\beta_{BNEW}=1\times 10^{-4}$, ε=0.85.

**Figure 3:**
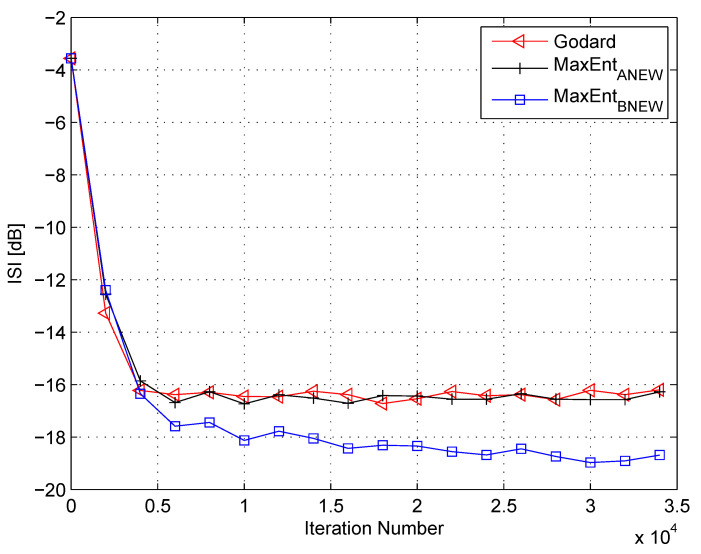
Performance comparison between equalization algorithms for a 16QAM source input going through Channel 1. The averaged results were obtained in 50 Monte Carlo trials for an SNR = 7 dB. $\mu_{G}=2.5\times 10^{-5}$, $\mu_{ANEW}=0.00008$, $\beta_{ANEW}=1\times 10^{-5}$, $\mu_{BNEW}=0.00008$, $\beta_{BNEW}=1\times 10^{-4}$, ε=0.85.

**Figure 4:**
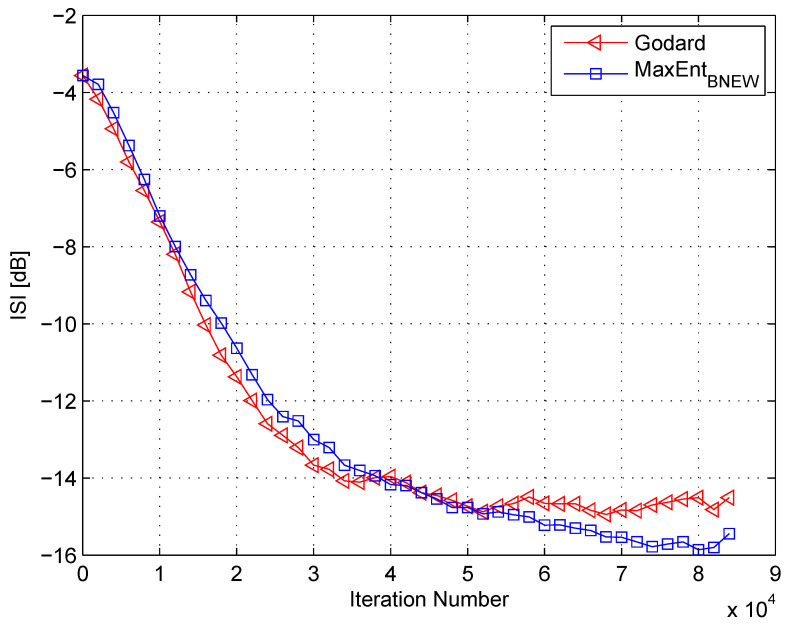
Performance comparison between equalization algorithms for a 16QAM source input going through Channel 1. The averaged results were obtained in 50 Monte Carlo trials for an SNR = 0 dB. $\mu_{G}=4\times 10^{-6}$, $\mu_{BNEW}=0.00002$, $\beta_{BNEW}=6\times 10^{-5}$, ε=0.05.

**Figure 5:**
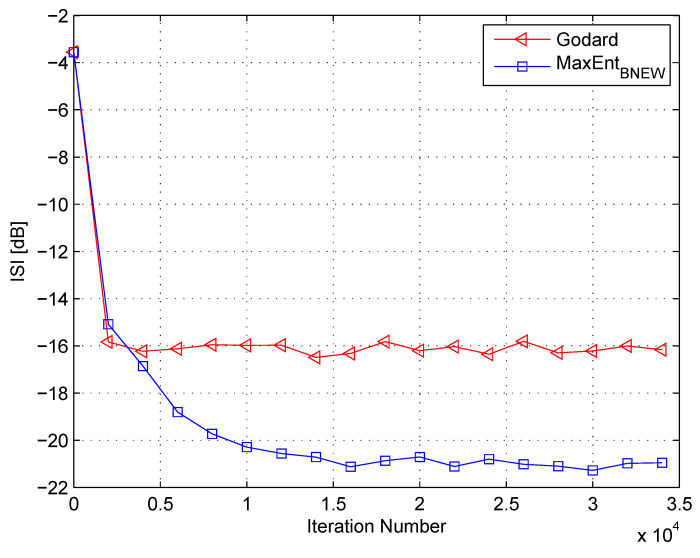
Performance comparison between equalization algorithms for a 16QAM source input going through Channel 2. The averaged results were obtained in 50 Monte Carlo trials for an SNR = 7 dB. $\mu_{G}=2.5\times 10^{-5}$, $\mu_{BNEW}=0.00008$, $\beta_{BNEW}=1\times 10^{-4}$, ε=0.85.

**Figure 6:**
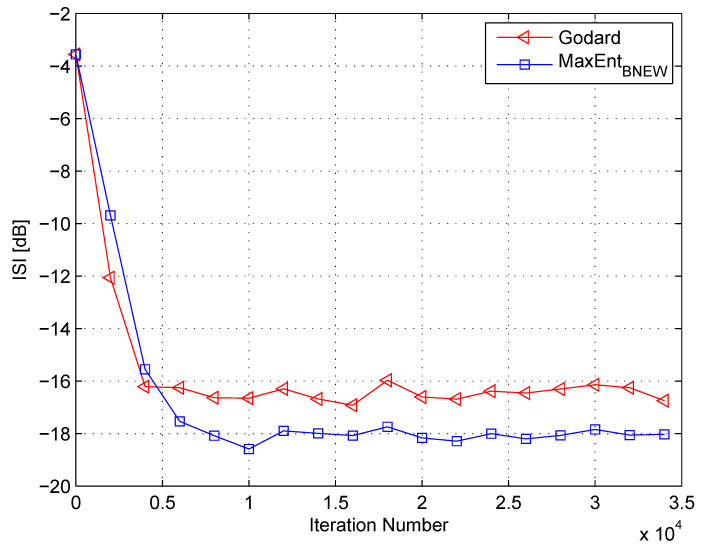
Performance comparison between equalization algorithms for a 16QAM source input going through Channel 3. The averaged results were obtained in 50 Monte Carlo trials for an SNR = 7 dB. $\mu_{G}=2.5\times 10^{-5}$, $\mu_{BNEW}=0.00007$, $\beta_{BNEW}=2\times 10^{-5}$, ε=0.85.

In my simulation, the equalizer’s length was set to 13 taps. For initialization purposes, the center tap of the equalizer was set to one while all others were set to zero. As a source input, I used the 16QAM constellation. The equalization performance comparison between the new proposed equalization method (“$MaxEnt_{BNEW}$” (46)), the maximum entropy [49], and Godard’s [53] algorithm is given in Figure 2, Figure 3 and Figure 4. The equalization performance comparison was carried out for a 16QAM constellation input sent via Channel 1 with SNR values of 10 dB, 7 dB, and 0 dB, respectively. It should be pointed out that the results in Figure 2 and Figure 3 for “$MaxEnt_{ANEW}$” and “Godard” were reproduced from Reference [49]. In addition, please note that according to Reference [49], “$MaxEnt_{ANEW}$” is not applicable for SNR=0 dB. According to Figure 2, Figure 3 and Figure 4, the new proposed algorithm (“$MaxEnt_{BNEW}$” (46)) has a better equalization performance from the residual ISI point of view compared to “$MaxEnt_{ANEW}$” and “Godard”. Next, I tested the proposed equalization method (“$MaxEnt_{BNEW}$” (46)) with Channel 2 and Channel 3 where $\sum_{k}{\left|h_{k}\right|}^{2}\neq 1$. Figure 5 and Figure 6 show the equalization performance comparison between the new proposed equalization method (“$MaxEnt_{BNEW}$” (46)) and Godard’s [53] algorithm for the 16QAM constellation input sent via Channel 2 and Channel 3, respectively, with SNR=7 dB. According to Figure 5 and Figure 6, the new proposed algorithm (“$MaxEnt_{BNEW}$” (46)) has a better equalization performance from the residual ISI point of view compared to “Godard”. As a matter of fact, for the case of $\sum_{k}{\left|h_{k}\right|}^{2}>1$ (Channel 2), the improvement in the residual ISI compared to the results obtained by “Godard” is approximately 5 dB while the improvement in the residual ISI compared to the results obtained by “Godard” for $\sum_{k}{\left|h_{k}\right|}^{2}<1$ (Channel 3) is only approximately 2 dB. Thus, we may say that the proposed algorithm (“$MaxEnt_{BNEW}$” (46)) has a promising equalization performance from the residual ISI point of view for channels with $\sum_{k}{\left|h_{k}\right|}^{2}\geq 1$.

Up to now, I have assumed that the SNR as well as the channel power are known. Thus, we could calculate the required Lagrange multipliers via (48). When the SNR and the channel power are unknown, the ${\hat{m}}_{2}$, ${\hat{m}}_{4}$, and ${\hat{m}}_{6}$ cannot be calculated anymore via (48). However, on the basis of (33), we may calculate the required ${\hat{m}}_{2}$, ${\hat{m}}_{4}$, and ${\hat{m}}_{6}$ needed for the Lagrange multipliers in (48) from

(53) $$\begin{array}{c} {\hat{m}}_{2}={\bar{m}}_{2}+\sigma_{{\tilde{w}}_{r}}^{2},\\ {\hat{m}}_{4}=3\sigma_{{\tilde{w}}_{r}}^{4}+6{\bar{m}}_{2}\sigma_{{\tilde{w}}_{r}}^{2}+{\bar{m}}_{4},\\ {\hat{m}}_{6}=15\sigma_{{\tilde{w}}_{r}}^{6}+45\sigma_{{\tilde{w}}_{r}}^{4}{\bar{m}}_{2}+15\sigma_{{\tilde{w}}_{r}}^{2}{\bar{m}}_{4}+{\bar{m}}_{6}, \end{array}$$

where $\sigma_{{\tilde{w}}_{r}}^{2}$ is the variance of the real part of $\tilde{w}\left[n\right]$ and may be simulated as

(54) $$\sigma_{{\tilde{w}}_{r}}^{2}=\sigma_{p_{1}}^{2}.$$

**Figure 7:**
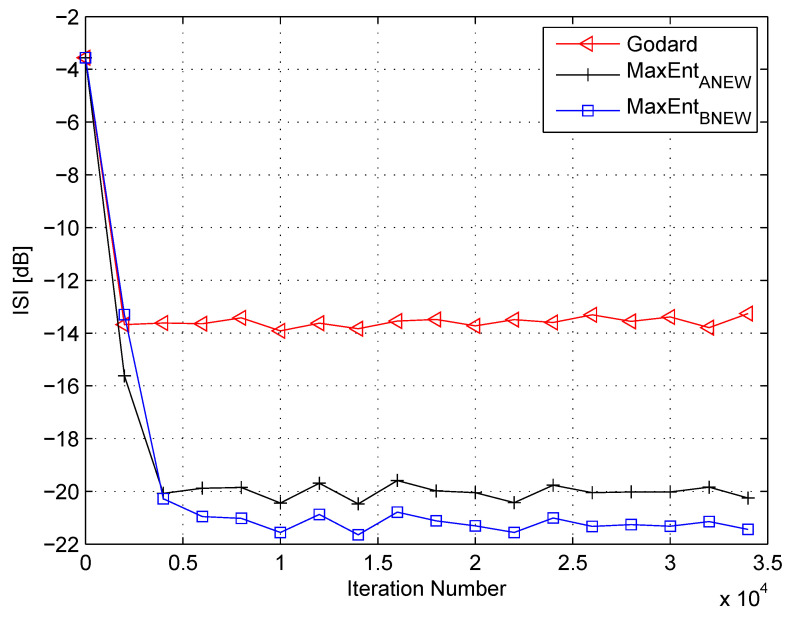
Performance comparison between equalization algorithms for a 16QAM source input going through Channel 1. The averaged results were obtained in 50 Monte Carlo trials for an SNR = 10 dB. $\mu_{G}=7\times 10^{-5}$, $\mu_{ANEW}=0.00009$, $\beta_{ANEW}=1\times 10^{-5}$, $\mu_{BNEW}=0.00007$, $\beta_{BNEW}=7\times 10^{-6}$, ε=0.75.

**Figure 8:**
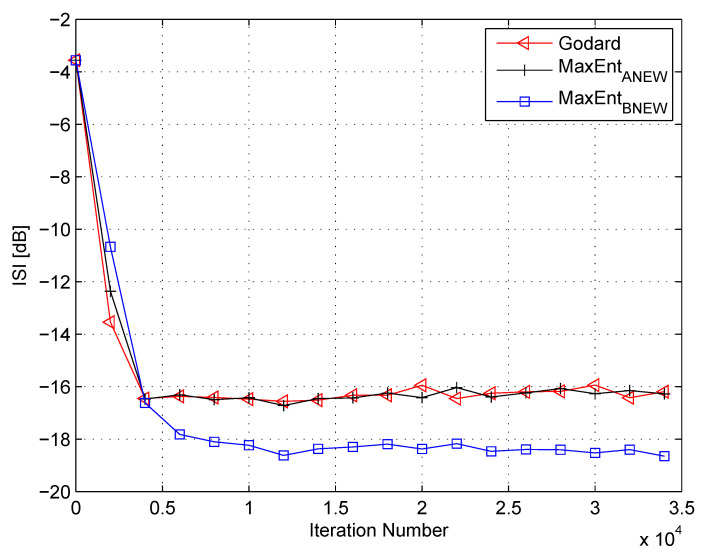
Performance comparison between equalization algorithms for a 16QAM source input going through Channel 1. The averaged results were obtained in 50 Monte Carlo trials for an SNR = 7 dB. $\mu_{G}=2.5\times 10^{-5}$, $\mu_{ANEW}=0.00008$, $\beta_{ANEW}=1\times 10^{-5}$, $\mu_{BNEW}=0.00006$, $\beta_{BNEW}=6\times 10^{-6}$, ε=0.75.

Please note that for the ideal case, when the equalizer has converged, the convolutional noise power tends to zero. Thus, this makes (54) reasonable. In the following, I use (53) and (54) for calculating the Lagrange multipliers related to the “$MaxEnt_{BNEW}$” algorithm. Figure 7 and Figure 8 show the equalization performance of the new proposed equalization method (“$MaxEnt_{BNEW}$” (46) with (53) and (54)), namely the ISI as a function of iteration number for the 16QAM constellation input sent via Channel 1 for SNR values of 10 dB and 7 dB, respectively, compared to the equalization performance obtained from the maximum entropy [49] and Godard’s [53] algorithm. Please note that here the results for “$MaxEnt_{ANEW}$” and “Godard” were also reproduced from Reference [49]. According to Figure 7 and Figure 8, the new proposed algorithm (“$MaxEnt_{BNEW}$” (46) with (53) and (54)) has better equalization performance from the residual ISI point of view compared to the maximum entropy [49] and Godard’s [53] algorithm even when the SNR and channel power are unknown.

## 5. Conclusions

In this paper, I proposed a new closed-form approximated expression for the conditional expectation (with Lagrange multipliers up to order four) which is actually a truncated version of the expression obtained in Reference [20]. This new proposed expression has a reduced computational burden compared with the previously obtained expressions for the conditional expectation proposed in References [20,47,49]. In addition, I derived new approximated closed-form expressions for the Lagrange multipliers ($\lambda_{2}$, $\lambda_{4}$). Simulation results have shown that my newly proposed equalization algorithm, with my newly proposed expression for the conditional expectation and Lagrange multipliers up to order four, is applicable for SNR values down to 0 dB and has an improved equalization performance from the residual ISI point of view compared with References [49,53].
